# Diffusion tensor imaging and voxel-based morphometry reveal corticospinal tract involvement in the motor dysfunction of adult-onset myotonic dystrophy type 1

**DOI:** 10.1038/s41598-018-34048-9

**Published:** 2018-10-22

**Authors:** Jin-Sung Park, Huijin Song, Kyung Eun Jang, Hyunsil Cha, Sang-Hoon Lee, Su-Keong Hwang, Donghwi Park, Hui Joong Lee, Jun-Young Kim, Yongmin Chang

**Affiliations:** 1 Department of Neurology, School of Medicine, Kyungpook National University, Daegu, Korea; 2 Department of Neurology, Kyungpook National University Chilgok Hospital, Daegu, Korea; 3 Institute of Biomedical Engineering Research, Kyungpook National University, Daegu, Korea; 4 Department of Medical & Biological Engineering, Kyungpook National University, Daegu, Korea; 5 Department of Pediatrics, School of Medicine, Kyungpook National University, Daegu, Korea; 6 Department of Rehabilitation, Daegu Fatima Hospital, Daegu, Korea; 7 Department of Radiology, Kyungpook National University Hospital, Daegu, Korea; 8 Department of Radiology, School of Medicine, Kyungpook National University, Daegu, Korea; 9 Department of Orthopaedic Surgery, Daegu Catholic University College of Medicine, Daegu, Korea; 10 Department of Molecular Medicine, School of Medicine, Kyungpook National University, Daegu, Korea

## Abstract

Magnetic resonance imaging (MRI) studies have demonstrated that patients with myotonic dystrophy type 1 (DM1) exhibit gray and white matter abnormalities that are correlated with various genetic and neuropsychological measures. However, few MRI studies have focused on the correlations between brain abnormalities and overall motor function including gait performance. Here, we investigated the correlations between brain abnormalities, as assessed with MRI including diffusion tensor imaging (DTI), and motor performance, as assessed with the Medical Research Council sum score (MRCSS), 6-minute walk test (6MWT), and hand grip power, in patients with DM1. Eighteen patients with DM1 and twenty healthy controls participated in this study. The MRCSS and 6MWT reflect patients’ general motor performance, particularly gait, while hand grip reflects the presence of myotonia. We found significant relationships between DTI parameters in the corticospinal tract (CST) and genetic factors and motor performance in patients with DM1. These findings suggest that CST involvement reflecting deterioration of the motor tracts may play a significant role in clinical myotonia. Further, a direct relationship between the cortical gray matter volume and DTI measures in the CST suggests that white matter abnormalities in the CST are strongly associated with volume reductions in the sensorimotor cortex of patients with DM1.

## Introduction

Myotonic dystrophy type 1 (DM1; OMIM: #160900) is a multisystem disorder that affects the muscles, eyes, heart, endocrine system, and central nervous system. It is caused by the abnormal expansion of unstable CTG trinucleotide repeats in the 3′-untranslated region of the myotonic dystrophy protein kinase gene (*DMPK*), which results in the intranuclear accumulation of mutated and mis-spliced transcripts, leading to an RNA gain of toxic function^1,2^. Furthermore, recent pathological studies have shown the presence of neurofibrillary tangles, which may explain the brain abnormalities that are observed in patients with DM1^3–5^.

Many previous magnetic resonance imaging (MRI) studies have revealed the presence of brain abnormalities in patients with DM1, including abnormalities in brain volume, cortical thickness, and white matter; several studies have also found that the structural abnormalities in gray and white matter were correlated with patients’ clinical and neuropsychological data^6–17^. However, most of these previous MRI studies focused on the correlations between cognitive or neuropsychological impairments and abnormalities in gray and white matter. Although a few studies demonstrated possible correlations between brain abnormalities and motor performance in DM1^12,16^, the specific relationship between brain abnormalities and overall motor function in DM1 has not been fully investigated. A few studies have attempted to show a relationship between motor function and brain abnormalities based on motor weakness or myotonia^12,17^. The novelty of our study is that we simultaneously evaluated the motor performance based on both motor weakness and gait performance; thus, our approach reflects the overall cardiopulmonary system in these patients.

In the current study, we investigated the correlations between brain abnormalities, as assessed with voxel-wise quantitative MRI (including diffusion tensor imaging (DTI) methods such as cortical volume analysis, tract-based spatial statistics (TBSS), and segmented tract analysis), and motor performance, as evaluated with the Medical Research Council sum score (MRCSS), 6-minute walk test (6MWT), and hand grip power, in patients with DM1, relative to age-matched healthy controls. The MRCSS and 6MWT reflect a patient’s general motor performance, with an emphasis on gait, while the hand grip power test assesses a patient’s ability to relax voluntary muscles after vigorous effort, a function that is often significantly deteriorated in patients with DM1; this aspect presents clinically as myotonia. Specifically, our aims were to (1) investigate the relationships between white and gray matter abnormalities (especially in the corticospinal tract (CST)) and various genetic and clinical characteristics of patients with DM1, particularly motor performance; and (2) evaluate the possible relationship between changes in cortical gray matter volume and DTI measurements of the CST in patients with DM1, in order to establish whether the abnormalities in gray matter and white matter are independent processes.

## Results

### Clinical and genetic parameters

The mean age of eighteen patients with DM1 was 44.22 (standard deviation [SD], 10.61) years, and the mean age of twenty healthy controls was 46.50 (SD, 9.84) years. For patients with DM1, the mean age at onset was 27.9 (11.8) years. The number of CTG repeats ranged from 150 to 778, and the mean CTG length was 360 (215). Patients’ hand grip score, according to the modified Rankin scale, ranged from 3 to 5, with the mean score being 3.61 (0.98). The mean disease duration was 16.3 (7.8) years.

### Relationship between gray matter volume and genetic and clinical measures

Table 1 shows the gray matter regions that were correlated with the genetic and clinical measures (CTG repeats, hand grip, and disease duration) of patients with DM1, as well as the results of the gray matter volume comparisons between DM1 and healthy controls. We found that CTG repeats were significantly correlated with the bilateral middle occipital and lunate sulci, right lateral orbital sulcus, and right posterior ramus of the lateral sulcus. These gray matter regions exhibited significant volume reductions in DM1 compared to healthy controls.

**Table 1:** Between-group comparison of gray matter volume and correlations between gray matter volume and genetic and clinical measures.

Clinical data	Gray matter region	Hemi- sphere	r-value (p-value)	Volume^a^ (SE)		P-value
				HC	DM1	
CTG repeat	Middle occipital sulcus and lunatus sulcus	L	−0.633 (0.005)**	1.426 (0.08)	1.154 (0.06)	0.008**
		R	−0.528 (0.024)*	1.297 (0.06)	1.024 (0.07)	0.005**
	Lateral orbital sulcus	R	−0.563 (0.015)*	0.638 (0.04)	0.478 (0.03)	0.002**
	Posterior ramus of the lateral sulcus	R	−0.530 (0.024)*	1.878 (0.05)	1.714 (0.05)	0.018*
Hand grip	Opercular part of the inferior frontal gyrus	L	0.568 (0.014)*	2.863 (0.08)	2.947 (0.16)	0.632
	Triangular part of the inferior frontal gyrus	L	0.731 (0.001)**	2.393 (0.08)	2.249 (0.12)	0.326
	Superior occipital gyrus	L	0.652 (0.003)**	2.484 (0.10)	1.916 (0.08)	<0.001**
	Superior part of the precentral sulcus	L	0.485 (0.041)*	2.147 (0.12)	1.697 (0.08)	0.004**
	Parcentral lobule and sulcus	R	0.491 (0.038)*	2.200 (0.07)	2.002 (0.10)	0.102
	Postcentral gyrus	R	0.493 (0.038)*	3.399 (0.10)	2.985 (0.13)	0.013*
	Anterior transverse temporal gyrus	R	0.589 (0.010)*	0.749 (0.04)	0.623 (0.04)	0.031*
	Inferior segment of the circular sulcus of the insula	R	0.546 (0.019)*	1.912 (0.05)	1.745 (0.06)	0.028*
	Middle occipital sulcus and lunatus sulcus	R	0.471 (0.048)*	1.297 (0.06)	1.024 (0.07)	0.005*
	Caudate	L	0.638 (0.004)**	3.343 (0.09)	3.215 (0.13)	0.423
		R	0.537 (0.022)*	3.525 (0.09)	3.435 (0.14)	0.580
	Amygdala	L	0.477 (0.045)*	1.634 (0.06)	1.374 (0.05)	0.002**
	Accumbens	L	0.511 (0.030)*	0.633 (0.03)	0.452 (0.03)	<0.001**
Disease duration	Brain stem		−0.598 (0.009)**	18.651 (0.43)	17.014 (0.37)	0.007**

^a^Unit of volume: ml; * and **indicate *p* < 0.01 and *p* < 0.05, res*p*ectively.

Patients’ hand grip scores were significantly correlated with various cortical gray matter regions including several limbic structures. Specifically, DM1 showed significant volume reductions in the left superior occipital gyrus, left superior part of the precentral sulcus, right postcentral gyrus, right anterior transverse temporal gyrus, right inferior segment of the circular sulcus of the insula, right middle occipital and lunate sulci, left amygdala, and left accumbens compared to healthy controls. The brainstem was correlated with disease duration and exhibited reduced volume in patients with DM1 vs. healthy controls (Table 1).

### Voxel-wise group comparisons of DTI data and correlations with genetic and clinical factors

**Figure 1:**
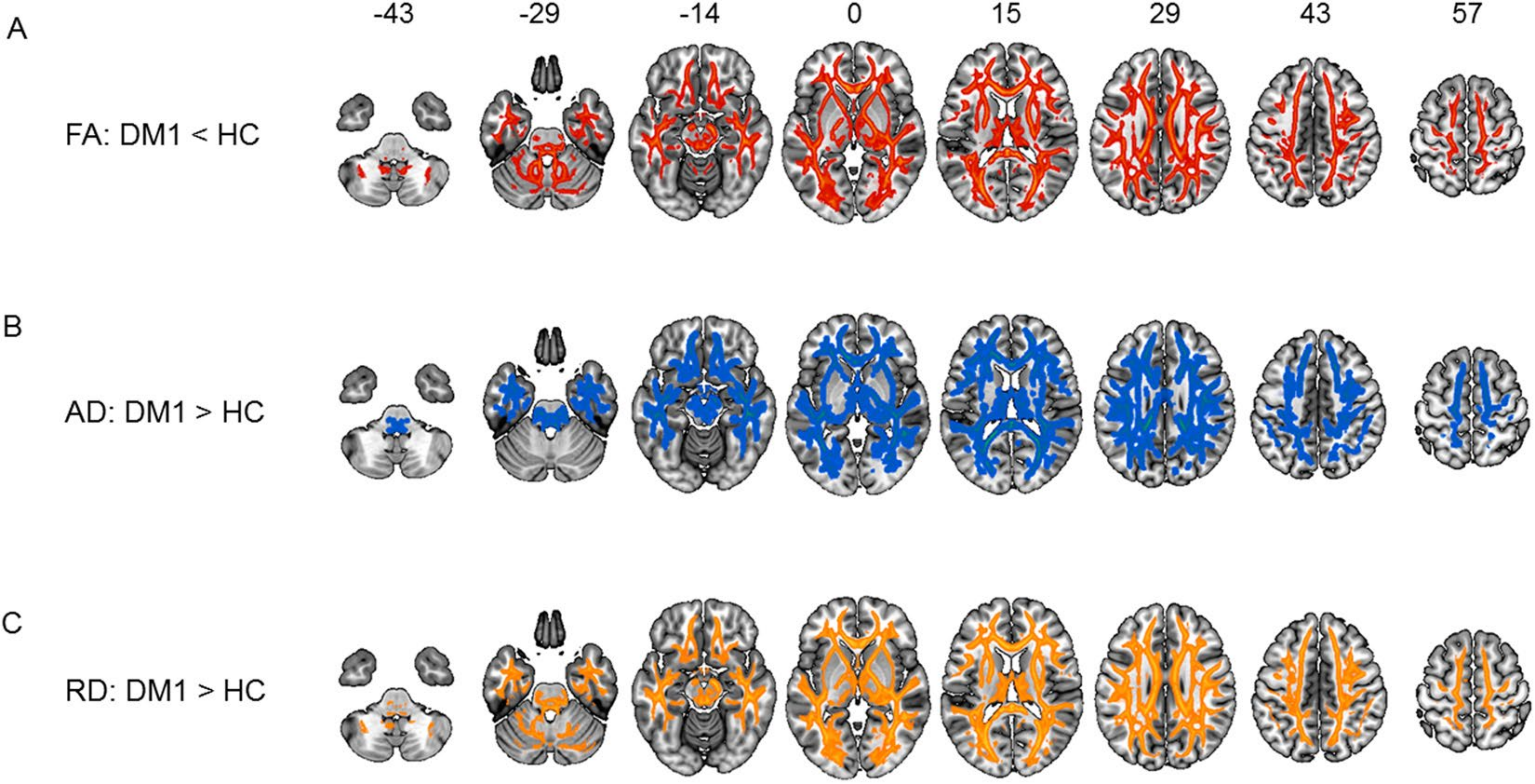
Between-group comparison of TBSS. Data are presented at p < 0.05 corrected for multiple comparisons. Voxel-wise differences show (**A**) reduced FA (red-yellow map), (**B**) elevated AD (blue-green map), and (**C**) elevated RD (orange map) in patients with DM1. TBSS = tract-based spatial statistics; FA = fractional anisotropy; AD = axial diffusivity; RD = radial diffusivity; DM1 = myotonic dystrophy type 1.

The DTI data were subjected to TBSS, which reflects the structural integrity of the white matter. The results identified widespread reductions in fractional anisotropy (FA) and elevations in axial diffusivity (AD) and radial diffusivity (RD) in DM1 compared to healthy controls (Fig. 1). The CTG repeats were significantly correlated with the posterior limb of the internal capsule (IC) and middle section of the CST (Table 2). Furthermore, the FA values in these motor-associated brain regions (posterior limb of the IC and middle section of the CST) showed strong correlations with patients’ performance on both the MRCSS and 6MWT, which reflect the motor function of patients with DM1 (Table 2). Significant differences in the DTI measures (FA, AD, and RD) of the posterior limb of the IC and middle section of the CST, which are important for motor function, were also identified between patients and healthy controls (Table 3).

**Table 2:** Correlations between white matter regions and genetic and clinical measures.

Clinical data	White matter regions	Hemi-sphere	r-value (p-value)		
			FA	AD	RD
CTG repeat	Posterior limb of IC	L/R	−0.682 (0.002)**	N/A	N/A
	Middle section of CST	L	−0.636 (0.005)**	−0.601 (0.008)**	0.553 (0.017)*
		R	−0.621 (0.006)**	−0.668 (0.002)*	0.543 (0.020)*
Hand grip	Midbrain section of CST	L	−0.603 (0.008)**	−0.669 (0.002)**	N/A
	Middle section of CST	L	0.493 (0.038)*	N/A	−0.555 (0.017)*
	Medial section of FMINOR	L/R	0.514 (0.029)*	N/A	N/A
Disease duration	Right middle section of FMINOR	R	−0.535 (0.022)*	N/A	N/A
MRCSS	Posterior limb of IC	L/R	0.735 (0.001)**	N/A	−0.474 (0.047)*
	Middle section of CST	R	0.640 (0.004)**	0.542 (0.020)*	−0.574 (0.013)*
6MWT	Posterior limb of IC	L	0.767 (0.001)**	N/A	−0.569 (0.034)*
	Middle section of CST	R	0.632 (0.021)*	N/A	N/A

FA = fractional anisotropy; AD = axial diffusivity; RD = radial diffusivity; IC = internal capsule; CST = corticospinal tract; FMINOR = forceps minor of the corpus callosum; * and **indicate *p* < 0.01 and *p* < 0.05, respectively.

**Table 3:** Between-group comparisons of the diffusion parameters in white matter regions and their correlations with genetic and clinical data.

White matter regions	Hemi-sphere	Diffusion parameter	Diffusion values(SE) of each group		P-value
			HC	DM1	
Posterior limb of IC	L/R	FA	0.3627 (0.0022)	0.3113 (0.0065)	<0.001**
		AD	0.00161 (0.00003)	0.00168 (0.00004)	0.109
		RD	0.00088 (0.00003)	0.00101 (0.00003)	0.005**
Upper section of CST	L	FA	0.4492 (0.0123)	0.4304 (0.0116)	0.275
		AD	0.00111 (0.00001)	0.00116 (0.00001)	0.002**
		RD	0.00054 (0.00001)	0.00058 (0.00000)	0.006**
Middle section of CST	L	FA	0.5914 (0.0138)	0.6087 (0.0116)	0.350
		AD	0.00119 (0.00001)	0.00123 (0.00001)	0.063
		RD	0.00044 (0.00001)	0.00044 (0.00001)	0.727
	R	FA	0.5213 (0.0067)	0.5046 (0.0124)	0.229
		AD	0.00115 (0.00001)	0.00123 (0.00002)	<0.001**
		RD	0.00047 (0.00000)	0.00052 (0.00000)	<0.001**
Midbrain section of CST	L	FA	0.5114 (0.0080)	0.5569 (0.0116)	0.002**
		AD	0.0012 (0.00002)	0.0013 (0.00002)	0.002**
		RD	0.00052 (0.00000)	0.00051 (0.00000)	0.056
Medial section of FMINOR	L/R	FA	0.7003 (0.0236)	0.6479 (0.0203)	0.069
		AD	0.00111 (0.00001)	0.00117 (0.00001)	0.002**
		RD	0.00046 (0.00005)	0.00055 (0.00004)	0.149

FA = fractional anisotropy; AD = axial diffusivity; RD = radial diffusivity; IC: internal capsule; CST: corticospinal tract; FMINOR = corpus callosum-forceps minor; * and ** indicate *p* < 0.01 and *p* < 0.05, res*p*ectively.

### Relationships between the diffusion parameters of the CST, forceps minor, and genetic and clinical measures

**Figure 2:**
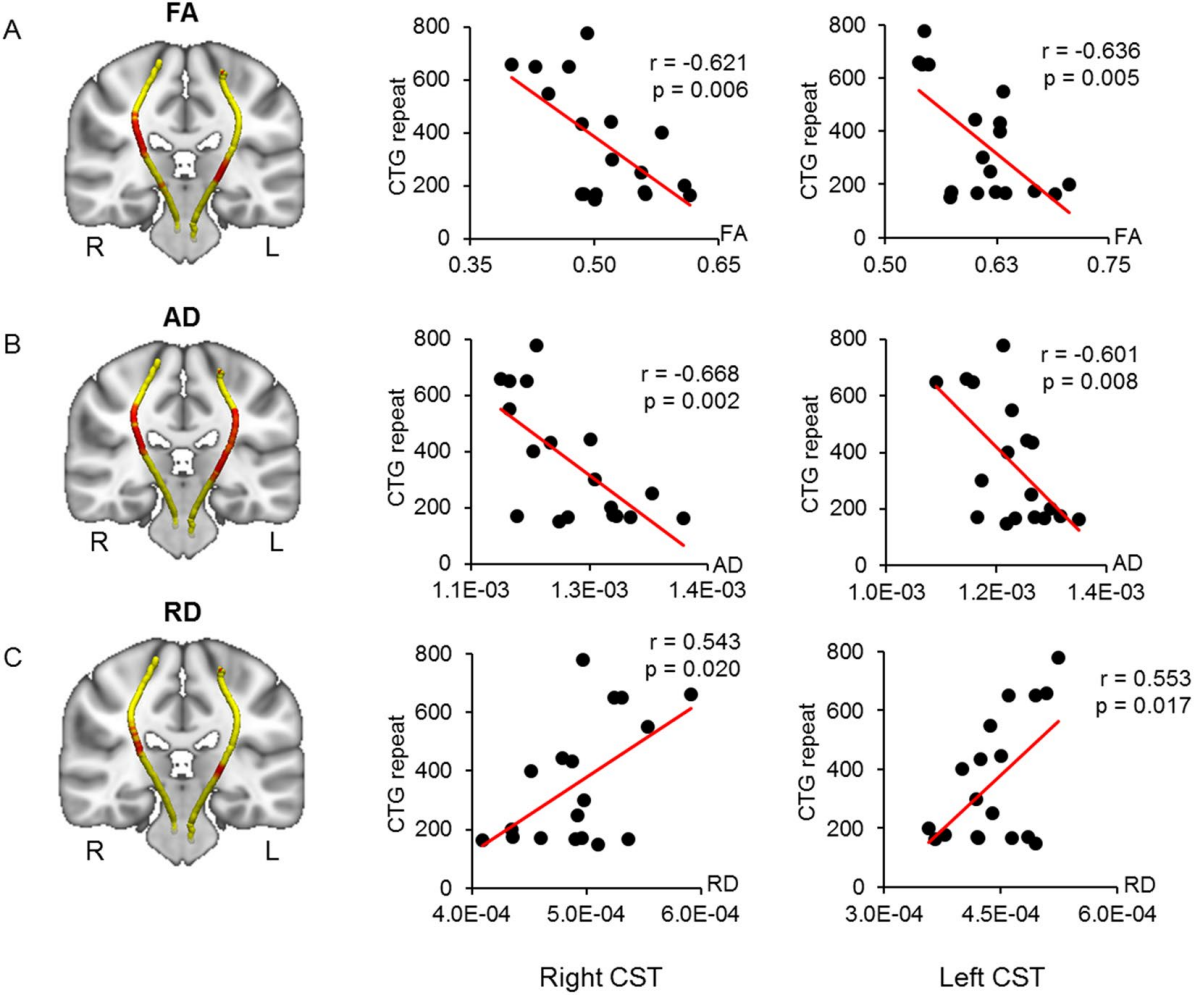
Correlated region projections and plots between the CST and CTG repeats. Relationships between the CST and CTG repeats are displayed projected onto a T1-weighted brain template and mean CST pathways (left panel). (**A,B**) Red regions in both CSTs show that CTG repeats are significantly correlated with reduced FA and AD (left panel). (**C**) Red regions show that the CTG repeats are significantly correlated with elevated RD in both CSTs (left panel). Middle and right panels show correlation plots for the red regions in both CSTs. CST = corticospinal tract; FA = fractional anisotropy; AD = axial diffusivity; RD = radial diffusivity.

Waypoint correlations between the CST and CTG repeats are shown in Fig. 2. The CTG repeats were negatively correlated with the FA and AD in the middle sections of the bilateral CST, which pass around the lateral ventricle and basal ganglia region (Fig. 2A,B). The CTG repeats were also positively correlated with the RD of the middle sections of the bilateral CST, though at fewer waypoints than the correlations with FA and AD (Fig. 2C). The right hemisphere of this region showed significantly elevated AD and RD values in patients with DM1 compared to healthy controls (Table 3). However, no significant group differences in the FA of the middle sections of CST in the right hemisphere or in the diffusion parameters (FA, AD, and RD) of the middle sections of CST in the left hemisphere were identified (Table 3).

**Figure 3:**
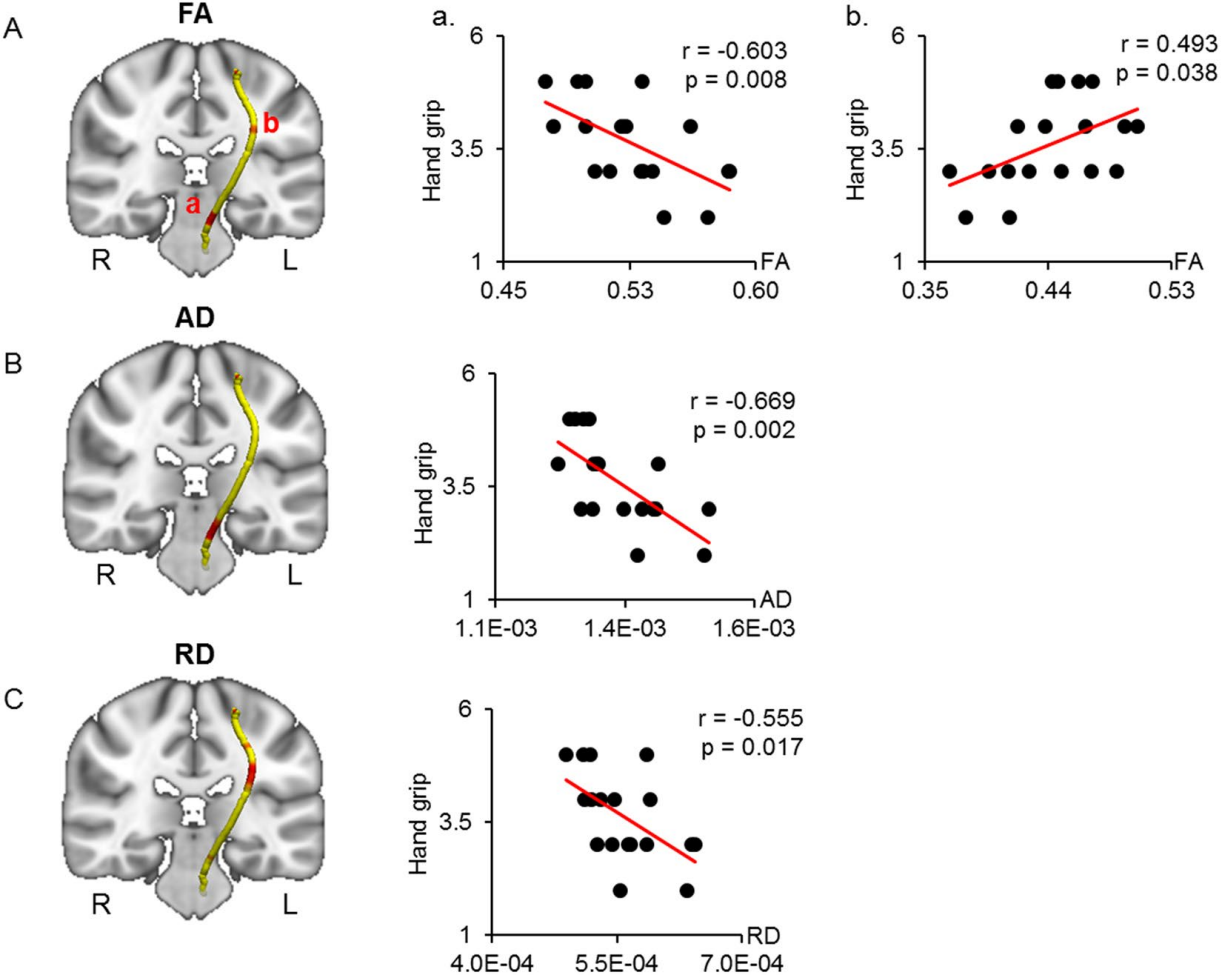
Correlated region projections and plots between the CST and hand grip. Relationships between the CST and hand grip are displayed projected onto a T1-weighted brain template and mean CST pathways (left panel). (**A**) Red regions in the left CST show significant correlations between FA and hand grip. Hand grip shows a significant correlation with reduced FA in the midbrain section of the left CST (a) and with elevated FA in the middle section of the left CST (b). (**B**) Red regions in the midbrain section of the left CST show a significant correlation between AD and hand grip. (**C**) Red regions in the middle section of the left CST show a significant correlation between RD and hand grip. CST = corticospinal tract; FA = fractional anisotropy; AD = axial diffusivity; RD = radial diffusivity.

Waypoint correlations between the CST and hand grip scores are shown in Fig. 3. The hand grip scores were negatively correlated with the FA and AD in the beginning sections of the left CST around the midbrain region (Fig. 3A,B). On the other hand, the FA and RD of the middle sections of the left CST were positively and negatively correlated with hand grip, respectively (Fig. 3A,C). Patients with DM1 showed significantly elevated FA and AD in the midbrain section of the left CST compared to healthy controls (Table 3). However, in the middle section of the left CST, only the AD value was elevated in DM1 compared to healthy controls (Table 3).

**Figure 4:**
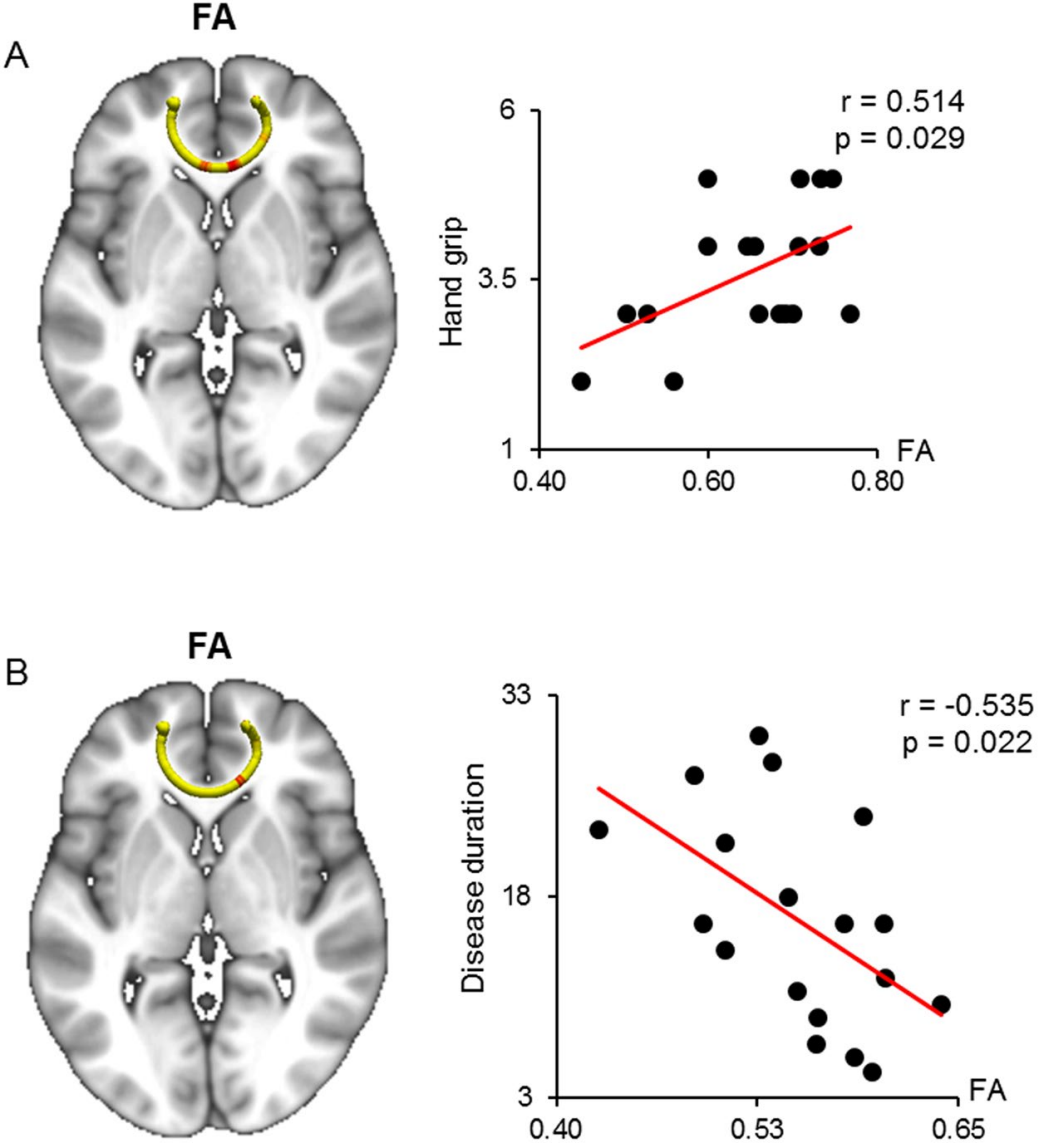
Correlated region projections and plots between the FMINOR and hand grip and disease duration. Relationships between the FMINOR and hand grip and disease duration are displayed on a T1-weighted brain template and mean FMINOR pathways (left panel). (**A**) Red regions show a significant correlation between FA and hand grip. Hand grip is significantly correlated with elevated FA in the red regions of the FMINOR. (**B**) Disease duration is significantly correlated with reduced FA in the red regions of the FMINOR. Right panels show correlation plots for the red regions of both CSTs, including Pearson’s coefficient and p-value. FMINOR = forceps minor of the corpus callosum; FA = fractional anisotropy.

Waypoint correlations between the diffusion parameters of the forceps minor (FMINOR) and hand grip and disease duration are shown in Fig. 4. Hand grip scores were positively correlated with the FA in the bilateral medial sections of the FMINOR (Fig. 4A). The AD of these regions was significantly elevated in patients with DM1 vs. healthy controls (Table 3). However, no significant differences in the FA and RD of the bilateral medial sections of the FMINOR were identified between the groups (Table 3). The disease duration was negatively correlated with the FA in the right middle section of the FMINOR (Fig. 4B). The AD of this region was significantly elevated in patients with DM1 vs. healthy controls, though no significant differences in the FA and RD of the right middle section of the FMINOR were noted between the groups (Table 3). The hand grip scores and disease duration were not significantly correlated with the AD and RD measures of the other waypoints of the FMINOR (Table 2).

**Figure 5:**
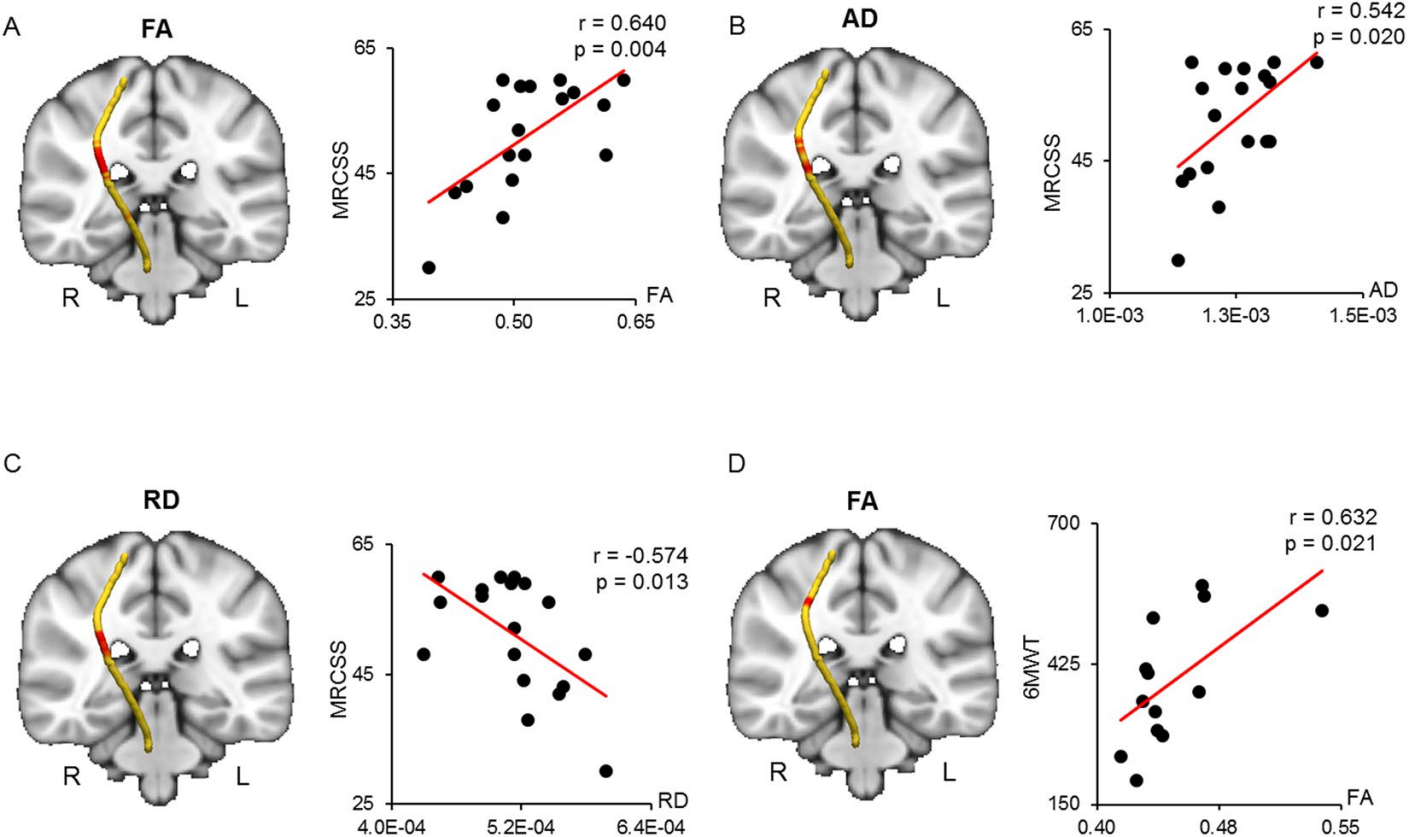
Correlated region projections and plots between the CST and MRCSS and 6MWT. Relationships between the right CST and MRCSS and 6MWT are displayed on a T1-weighted brain template and mean right CST pathway (left panel). (**A**) Red regions in the right CST show a significant correlation between FA and MRCSS. (**B**) The MRCSS is significantly correlated with elevated AD in the red regions of the right CST. (**C**) The MRCSS is significantly correlated with reduced RD in the red regions of the right CST. (**D**) The 6MWT is significantly correlated with elevated FA in the red regions of the right CST. CST = corticospinal tract; MRCSS = Medical Research Council sum score; 6MWT = 6-minute walk test; FA = fractional anisotropy; AD = axial diffusivity; RD = radial diffusivity.

Waypoint correlations between the CST and MRCSS and 6MWT are shown in Fig. 5. The MRCSS was positively correlated with the FA and AD in the middle section of the right CST (Fig. 5A,B). The RD of a similar region of the right CST was negatively correlated with the MRCSS (Fig. 5C). The AD and RD of the middle section of the right CST were significantly elevated in patients with DM1 compared to healthy controls (Table 3). No significant FA differences were noted between the groups in this region (Table 3). The 6MWT was positively correlated with only the FA in the upper region of the middle section of the right CST (Fig. 5D). In this region, patients with DM1 showed significantly reduced FA and elevated AD and RD compared to healthy controls (Table 3).

### Relationship between the CST diffusion parameters and motor cortex volume

**Figure 6:**
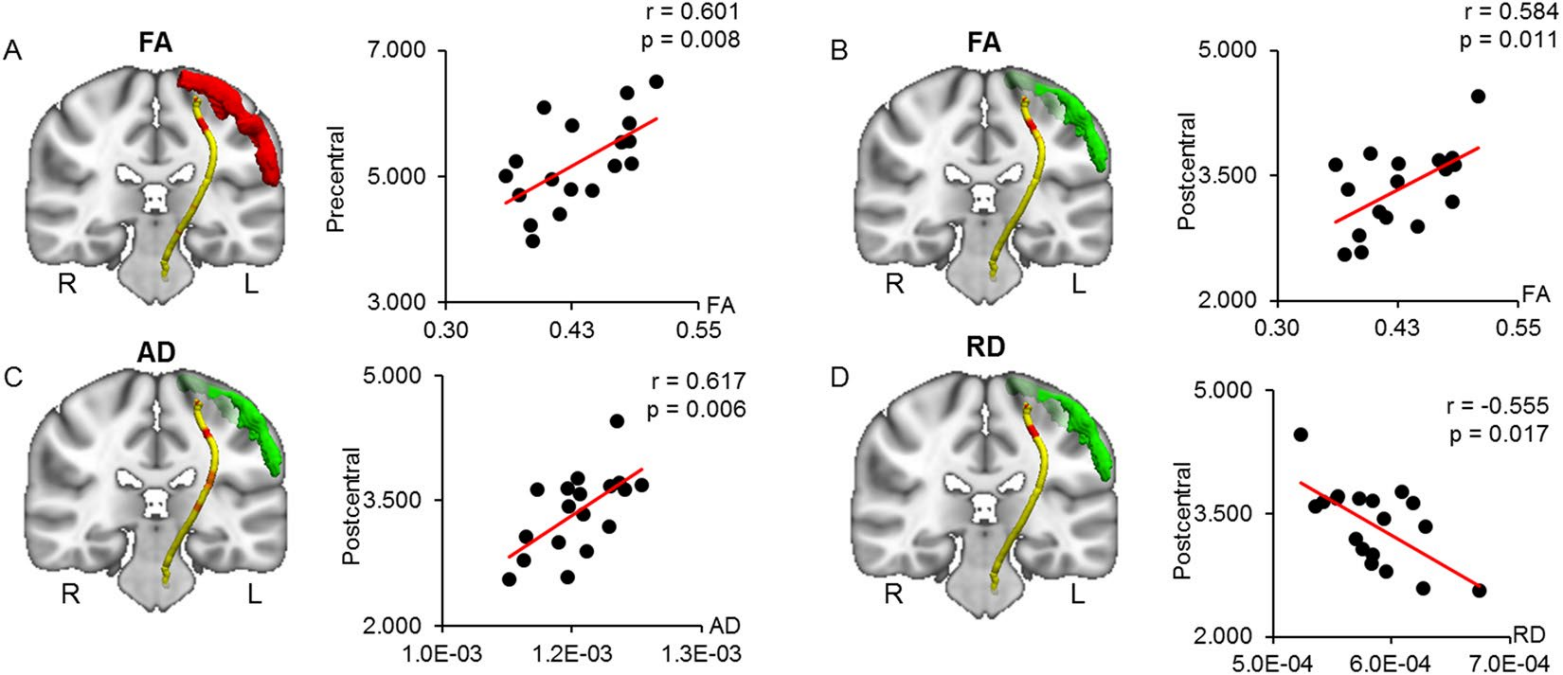
Correlated region projections and plots between the CST and precentral and postcentral gyri. Relationships between the CST and precentral and postcentral gyri volumes are displayed on a T1-weighted brain template and mean CST pathways. (**A**) Precentral gyrus volume (red) is significantly correlated with elevated FA in the red region of the left CST. (**B**,**C**) Postcentral gyrus volume (green) is significantly correlated with elevated FA and AD in the red regions of the left CST. (**D**) Postcentral gyrus volume (green) is significantly correlated with reduced RD in the red region of the left CST. FA = fractional anisotropy; AD = axial diffusivity; RD = radial diffusivity.

Waypoint correlations between the diffusion parameters of the CST and the volume of the precentral and postcentral gyri are shown in Fig. 6. The precentral gyrus volume was positively correlated with only the FA in the ending sections of the CST, which are close to the motor cortex (Fig. 6A). The postcentral gyrus volume was significantly correlated with the FA, AD, and RD in similar sections of the CST with waypoint correlations between the precentral gyrus and CST (Fig. 6B–D). The AD and RD in these regions were significantly elevated in DM1 vs. healthy controls (Table 3). However, the FA in these regions was shown no differences between the two groups.

## Discussion

The results from the current study are in accordance with those of previous studies, which have shown widespread brain abnormalities in patients with DM1. However, in contrast to previous imaging studies, which focused on structural abnormalities and their relationships with cognitive and neuropsychological impairments in DM1, the current study used DTI and TBSS to reveal strong relationships between the motor performance of patients with DM1 and abnormalities in the CST, which is the prototypic tract reflective of motor function. Furthermore, the present study showed that the gray matter volume in the sensorimotor cortex is correlated with alterations in DTI measurements of the CST, suggesting that cortical volume reductions in the sensorimotor cortex are associated with microstructural white matter abnormalities observed in the CST in patients with DM1. The current study used a methodological approach similar to that of previous literature^16^, comprising voxel-wise DTI comparison using TBSS (from FSL) and cortical thickness analysis using FreeSurfer; in this study, we added a fully automatic pipeline of FreeSurfer for both cortical thickness and volumetric analysis.

In accordance with the existing imaging literature^6–8,10,11,13–21^, we observed reduced gray matter volume in several brain regions in patients with DM1, compared with the volume in healthy controls. Among these brain regions, reduction of gray matter volume in the occipital area was significantly correlated with the number of CTG repeats in patients with DM1. CTG repeats comprise a known genetic factor reflective of disease severity^9^. More importantly, deterioration of hand grip in patients with DM1 was significantly correlated with reduction of gray matter volume in several brain regions that are important for motor performance (e.g., precentral and postcentral gyri). These findings imply that the reduction of gray matter volume in the sensorimotor cortex of individuals with DM1 likely affects their motor performance: with greater reduction of gray matter volume, worse hand grip performance is observed in patients with DM1.

DTI parameters in several sections of the CST were correlated with genetic and motor-associated clinical measures. First, CTG repeats were robustly correlated with DTI parameters (FA, AD, and RD) in the posterior limb of the IC and middle section of the CST, indicating that the white matter microintegrity of these portions of the CST exhibits the greatest susceptibility to genetic factors in patients with DM1. The posterior limb of the IC contains CST fibers, which arise in the motor area of the cerebral cortex, and sensory fibers, largely derived from the thalamus, which pass upward to the sensory cortex. Additionally, DTI parameters in the middle section of the CST were strongly correlated with motor-associated clinical measures. Specifically, FA values in the posterior limb of the IC and middle section of the CST were primarily correlated with patient performance on three clinical assessments of motor function (hand grip test, MRCSS, and 6MWT). Furthermore, RD values in the posterior limb of the IC and middle section of the CST were correlated with patient performance on these same clinical assessments of motor function, suggesting that the correlation between the clinical measures and FA may result from the correlation between the clinical measures and RD. Therefore, when considering FA as a function of RD^22^, it is tempting to interpret these results as indicative of RD as the main source of microstructural abnormalities along the CST in patients with DM1. More specifically, the elevation in RD, as observed in patients with DM1, relative to that of healthy controls, seems to indicate that demyelination of white matter occurs along the CST in patients with DM1^23,24^.

A recent study of 16 adolescent DM1 with a mean age of 13.9 found a significant difference in the DTI of the corticospinal tract; notably, the difference was present in MD, but not in FA^11^. Another study recruited 45 DM1 patients with a mean age of 38.4; they exhibited statistically significant differences in both FA and MD of the corticospinal tract, compared with controls^12^. The mean age of DM1 patients enrolled in the current study was 44.22; we found statistically significant differences in both FA and MD. Based on these findings, we can speculate on the contribution of disease progression to alterations in imaging findings; however, further longitudinal studies are needed to clarify this relationship.

A recent electrophysiological study showed a delay in the central motor conduction time in patients with DM1^25^, supporting our findings of abnormalities in the CST in patients with DM1. Clinically, DM1 is characterized by hand myotonia, which is a dysfunction of hand muscle relaxation. We suspect that, as greater effort is needed to extend the fingers, hyperexcitation may be induced in the CST; this may cause more extensive damage to the CST, compared with other tracts in the brain. Our hypothesis is supported by a recent fMRI study that showed enhancement of cerebral blood oxygen level-dependent (BOLD) signals in the supplementary motor area (SMA), which harbors the CST^26^. Therefore, early and significant involvement of the CST may be a consequence of reorganization and redistribution of brain circuits due to hyperexcitation, as individuals with clinical myotonia must use additional effort to accomplish the same motor task, compared with healthy controls.

Our study also revealed an association between white matter abnormalities in the CST and changes in gray matter volume in the sensorimotor cortex. Specifically, lower FA values were associated with greater volume reductions in the precentral and postcentral cortices. Furthermore, the postcentral cortical volume was positively correlated with the AD and negatively correlated with the RD in the terminal sections of the CST. This finding is important because it clarifies the relationship between gray matter and white matter abnormalities in DM1, indicating that they are not independent processes. Indeed, our findings suggest that in DM1, white matter abnormalities in the CST are strongly associated with reductions in gray matter volume in the sensorimotor cortex; the inverse relationship is also likely to exist. Prior studies have shown widespread atrophy of the corpus callosum in DM1; moreover, DTI images have shown a significant loss of volume in the corpus callosum, supporting the role of Wallerian degeneration as a major cause of white matter change in DM1^8,19^.

In conclusion, our results are in accordance with those of previous imaging studies that have demonstrated diffuse white and gray matter abnormalities in DM1. However, the current study revealed new statistically significant relationships between DTI parameters in the CST and genetic and motor-associated clinical characteristics in patients with DM1. These findings suggest that involvement of the CST, reflecting deterioration of the motor tract, may play a significant role in clinical myotonia. Moreover, the direct relationship we identified between cortical gray matter volume and DTI measures in the CST of patients with DM1 suggests that in these patients, white matter abnormalities in the CST are strongly associated with reductions in gray matter volume in the sensorimotor cortex. Finally, because of the relatively small population in this study, it is necessary to include a larger population to confirm the current findings in the future study.

## Methods

### Participants

Eighteen patients with DM1 (8 men, 10 women) and twenty healthy controls (9 men, 11 women) participated in this study. The mean age of the patients with DM1 was 44.22 (SD, 10.61) years and the mean age of the healthy controls was 46.50 (SD, 9.84) years. The clinical characteristics, number and length of CTG repeats, hand grip score, age of onset, disease duration, and laboratory findings including the creatine kinase level of the participants were obtained. The MRCSS test was applied to evaluate the motor function of the DM1 patients. The MRCSS was calculated by summing the MRC scores of six muscle groups (elbow flexion, abduction of shoulder, wrist extension, knee extension, hip flexion, and foot dorsiflexion for both sides). The MRC score for each muscle group ranged from 0 to 5, yielding a maximum score of 60^27^. We additionally used MRC scoring to determine the bilateral hand grip, as distal hand weakness is one of the early signs of DM1. Genomic DNA was isolated from the peripheral blood as described previously^28^. In addition, we employed the 6MWT, which represents motor function and cardiopulmonary function in DM1. The 6MWT was performed and scored according to the methods of a previous study^29^. All persons who were participated in the data analysis, were blinded to the identities of individual cases and presence of white matter abnormalities on conventional T2W or FLAIR images. Approval for all procedures was received from the Institutional Review Board of Kyungpook National University Medical Center, and written informed consent was obtained from all participants by the requirements of the Kyungpook National University Hospital Human Research Committee. In addition, all experiments were performed in accordance with relevant guidelines and regulations.

### Image acquisition

We acquired MRI data from all participants using a 3-T scanner (Discovery MR750, GE healthcare), with a 32-channel head coil. Structural brain images for the volumetric analyses were acquired using the three-dimensional brain volume imaging sequence (repetition time: 8.16 ms, echo time: 3.18 ms, flip angle: 12°, 1-mm isotropic resolution). We acquired DTI data using the diffusion-weighted echo planar imaging sequence, with 25 directions and a b-value of 1000 (repetition time: 10,000 ms, echo time: 100.2 ms, thickness: 4 mm, flip angle: 90°, 0.82-mm in-plane resolution).

### Cortical volumetric analysis

Structural images were processed using a fully automatic pipeline for cortical reconstruction and volumetric segmentation within the FreeSurfer 5.3 program (http://surfer.nmr.mgh.harvard.edu). Briefly, this procedure includes skull stripping, automated Talairach transformation, subcortical segmentation of white and gray matter, intensity normalisation, and tessellation of the gray/white matter boundary and pial surface by using continuity information and intensities image intensities from the structural volume. The Destrieux parcellation atlas^30^ was applied to automatically parcellate and assign a neuroanatomical label to each location on a cortical surface model. The surface of the gray and white matter border was inflated and smoothed with a 10-mm full width at half maximum Gaussian smoothing kernel. Each participant’s data were registered to an average spherical surface representation in Freesurfer (fsaverage). Manual editing was not performed at any stage of the Freesurfer processing stream. The volume data extracted by the parcellation process for each labelled region were employed in the correlation analysis with DTI and clinical data. Pearson’s correlation test was applied to estimate correlation coefficient between the volume data and clinical data. The two-sample t-test was used to compare differences of gray matter volumes, which extracted from each labelled region, between DM1 and healthy controls with the significant level of p < 0.05.

### Diffusion tensor data analysis

Using the DTI data, we performed TBSS, which is part of the FMRIB Software Library (FSL; http://fsl.fmrib.ox.ac.uk/fsl/fslwiki). Each participant’s diffusion data were preprocessed with eddy current correction tools in FSL, which correct for eddy current distortions and head motion. The b0 image from each participant was used as a reference image for realigning the diffusion data. Subsequently, a brain mask was created with the b0 image after automated skull stripping was performed using the brain extraction tool (BET). The brain mask was applied to perform diffusion tensor estimation using FMRIB’s diffusion toolbox. The FA, AD, and RD data for each brain voxel were calculated by the diffusion toolbox. The FA was calculated from the eigenvalues (*λ*_1_, *λ*_2_, *λ*_3_) of the diffusion tensor, as follows:

$${\rm{FA}}=\sqrt{\frac{3(({\lambda }_{1}\,-\bar{\lambda }{)}^{2}+{({\lambda }_{2}-\bar{\lambda })}^{2}+{({\lambda }_{3}-\bar{\lambda })}^{2})}{2\sqrt{{\lambda }_{1}^{2}+{\lambda }_{2}^{2}+{\lambda }_{3}^{2}}}},$$

where $\bar{\lambda }$ represents the mean value of the eigenvalues^31^. The AD, which reflects diffusivity of water along the direction of the axonal fibres (*λ*_1_), and the RD, termed (*λ*_1_ + *λ*_2_)/2, which reflects diffusivity of water perpendicular to the fibres, were also calculated using the eigenvalues^32^. Each individual FA data were registered to the FMRIB58 FA standard space using the FMRIB’s nonlinear image registration tool. A mean FA image was calculated with a minimum threshold of 0.2 and thinned to create skeletonized a mean FA, which represented the centres of all tracts common to all participants. Each individual’s aligned FA data were projected onto the skeleton and the resulting data were used to perform voxel-wise comparisons between the groups. The mean AD and RD data were processed with the same nonlinear registration and projection procedures as those described above.

A general linear model was used to test for between-group differences and correlations with clinical data. The randomise tool in FSL was used for multiple comparisons, with 5000 permutations of the data. Differences were considered significant at a family-wise error-corrected p value of <0.05. The threshold-free cluster enhancement method was used to define clusters for significant differences in between the groups. Correlation analyses were performed with clinical variables (CTG repeats, disease duration, MRCSS, and 6MWT) for voxel-wise correlation analysis within the DM1 group.

### Segmented tract analysis

For tract segmentation, the tracts constrained by underlying anatomy (TRACULA) program within FreeSurfer 5.3 was used for the DTI data processing. The TRACULA program is a processing stream that includes an algorithm for performing automated global probabilistic tractography to estimate the posterior probability of the following 18 major white matter tracts: CST, cingulum-cingulate gyrus bundle, cingulum angular bundle, inferior longitudinal fasciculus, superior longitudinal fasciculus-temporal part, longitudinal fasciculus-parietal part, anterior thalamic radiation, uncinate fasciculus, in each hemisphere; and the forceps major and FMINOR of the corpus callosum. First, the preprocessing stage performed eddy current compensation to correct for eddy currents and head movements and intra-subject and inter-subject registration to the FreeSurfer common template space by FreeSurfer’s bbregister; tensor fitting was also applied for least-squares tensor estimation using FSL’s dtifit. Second, the TRACULA program invoked FSL’s bedpostx tool to apply the ball-and-stick model of diffusion to the DTI data^33^. The TRACULA program then estimated the posterior probability of each pathway in the participant with a pathway prior information on each tract’s position combined with the participant’s anatomical segmentation labels. Finally, the TRACULA program combined the participant’s diffusion measures (FA, AD, and RD) along each pathway and output a table for each diffusion measure and each pathway. We used the diffusion measures from the FMINOR and CST of both hemispheres to investigate the relationships between motor function and clinical measures (CTG repeats, hand grip, MRCSS, 6MWT, and disease duration). For statistical test, Pearson’s correlation test was used to estimate correlation coefficient between diffusion data and clinical measures. The two-sample t-test was used to compare differences of diffusion parameters (FA, AD, and RD) for group comparison at significant level of p < 0.05.

### Statistical analysis

Statistical analyses were performed using SPSS version 22.0 (IBM). We tested the associations between the mean diffusion measures (FA, AD, and RD) of the CST and FMINOR, the extracted gray matter volume of each labelled region by FreeSurfer, and the genetic and clinical measures using Pearson’s correlation tests. The level of significance was set at p < 0.05 (two-tailed).

## Data Availability

The datasets generated during and/or analysed during the current study are available from the corresponding author on reasonable request.
